# (1*R*,2*R*)-*N*,*N*′-Bis(ferrocenylmeth­yl)-1,2-diphenyl­ethane-1,2-diamine

**DOI:** 10.1107/S1600536810024293

**Published:** 2010-07-21

**Authors:** Yi Guo, Jianfeng Wang, Chixiao Zhang, Shuping Luo

**Affiliations:** a State Key Laboratory Breeding Base of Green Chemistry-Synthesis Technology, Zhejiang University of Technology, Hangzhou 310014, People’s Republic of China

## Abstract

The title compound, [Fe_2_(C_5_H_5_)_2_(C_26_H_26_N_2_)], was synthesized from a chiral diamine and ferrocenecarboxaldehyde and subsequent reduction with NaBH_4_. It has two chiral centers which both exhibit an *R* configuration. Two ferrocene groups are present in the mol­ecular structure, with their cyclo­penta­dienyl ring planes showing an almost perpen­dicular arrangement [dihedral angle 88.6 (1)°].

## Related literature

For related compounds, see: Hess *et al.* (1999 ▶); Bâse *et al.* (2002 ▶); Wang (2009 ▶).
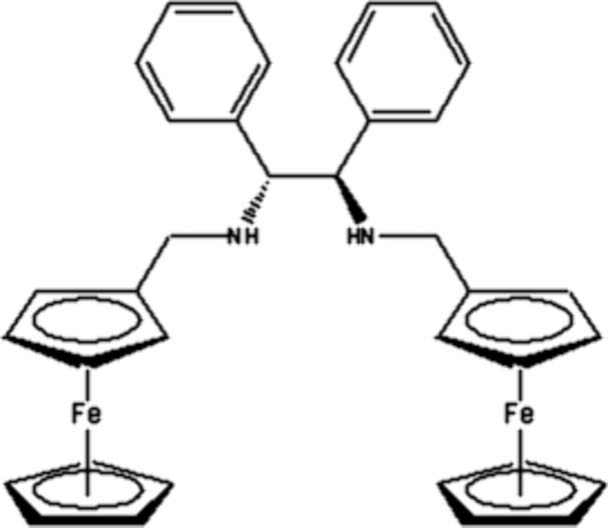


## Experimental

### 

#### Crystal data

[Fe_2_(C_5_H_5_)_2_(C_26_H_26_N_2_)]
*M* *_r_* = 608.37
Orthorhombic, 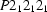

*a* = 9.5080 (2) Å
*b* = 9.5700 (2) Å
*c* = 32.3680 (7) Å
*V* = 2945.21 (11) Å^3^
*Z* = 4
Mo *K*α radiation
μ = 1.01 mm^−1^
*T* = 295 K
0.36 × 0.20 × 0.18 mm

#### Data collection

Rigaku R-AXIS RAPID/ZJUG diffractometer
Absorption correction: multi-scan (*ABSCOR*; Higashi, 1995 ▶) *T*_min_ = 0.712, *T*_max_ = 0.839
45235 measured reflections
6726 independent reflections
5515 reflections with *I* > 2σ(*I*)
*R*_int_ = 0.036

#### Refinement

*R*[*F* ^2^ > 2σ(*F* ^2^)] = 0.030
*wR*(*F* ^2^) = 0.077
*S* = 1.01
6726 reflections
361 parameters
30 restraints
H-atom parameters constrained
Δρ_max_ = 0.37 e Å^−3^
Δρ_min_ = −0.23 e Å^−3^
Absolute structure: Flack (1983 ▶), 2915 Friedel pairs
Flack parameter: −0.006 (14)

### 

Data collection: *PROCESS-AUTO* (Rigaku, 2006 ▶); cell refinement: *PROCESS-AUTO*; data reduction: *CrystalStructure* (Rigaku, 2007 ▶); program(s) used to solve structure: *SHELXS97* (Sheldrick, 2008 ▶); program(s) used to refine structure: *SHELXL97* (Sheldrick, 2008 ▶); molecular graphics: *ORTEP-3 for Windows* (Farrugia, 1997 ▶); software used to prepare material for publication: *WinGX* (Farrugia, 1999 ▶).

## Supplementary Material

Crystal structure: contains datablocks I, global. DOI: 10.1107/S1600536810024293/im2210sup1.cif

Structure factors: contains datablocks I. DOI: 10.1107/S1600536810024293/im2210Isup2.hkl

Additional supplementary materials:  crystallographic information; 3D view; checkCIF report

## Figures and Tables

**Fig. 1.**
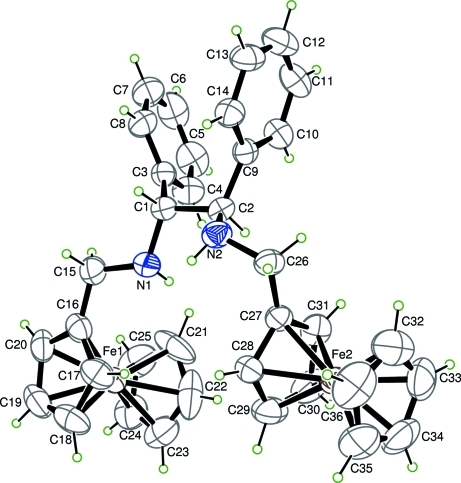
Molecular structure of the title compound, displacement ellipsoids are drawn at the 40% probability level.

## supplementary crystallographic information

### Comment

Due to the special structure and electronic properties of ferrocene, its derivatives have been widely used in material sciences, biology and asymmetric catalysis. The title compound, which could be readily synthesized from commercially available ferrocenecarboxaldehyde and a chiral diamine, is envisaged to potentially act as a chiral ligand in asymmetric catalysis.

The crystal structure of the title compound is shown in Fig. 1. It contains the (1*R*,2*R*)-(+)-1,2-diphenyl-1,2-ethanediamine subunit, meanwhile, the hydrogen atoms of the two amine groups have been substituted by ferrocenylmethyl units. The angle between the two benzene ring planes is 36.3 (1)°. The Cp ring planes (C21—C25 and C27—C31), are almost perpendicular to each other [dihedral angle 88.6 (1)°]. The intramolecular Fe—Fe distance is 7.064 Å.

### Experimental

A solution of ferrocenecarboxaldehyde (5 mmol, 1070 mg) in 20 ml absolute methanol was added dropwise to a stirred solution of (1*R*,2*R*)-(+)-1,2-diphenyl-1,2-ethanediamine (2.5 mmol, 530 mg) in absolute methanol (10 ml). After stirring for 12 h, the reaction mixture was filtered obtaining brown crude imine. The imine was added to 25 ml absolute methanol and 25 ml THF. Solid NaBH_4_ (1.5 g, 40 mmol) was added in small portions over a period of 1 h. The resulting clear solution was allowed to stir at room temperature overnight. The solvent was removed *in vacuo*. Then 100 ml of water were added, extracted with dichloromethane. The combined organic layer was concentrated under reduced pressure to afford the crude product which was purified by flash chromatography (silica gel, Hex/AcOEt, *v*/*v*, 9:1), giving a yellow solid (overall yield 73%). Single crystals were obtained by slow evaporation of a dichloromethane solution.

### Refinement

H atoms were placed in calculated position with N—H=0.86 Å, C—H=0.93–0.97 Å and were included in the refinement in the riding model with *U*_iso_(H) = 1.2U_eq_ of the carrier atoms.

### Crystal data

**Table:**

[Fe_2_(C_5_H_5_)_2_(C_26_H_26_N_2_)]	*F*(000) = 1272
*M**_r_* = 608.37	*D*_x_ = 1.372 Mg m^−^^3^
Orthorhombic, *P*2_1_2_1_2_1_	Mo *K*α radiation, λ = 0.71073 Å
Hall symbol: P 2ac 2ab	Cell parameters from 36065 reflections
*a* = 9.5080 (2) Å	θ = 3.0–27.4°
*b* = 9.5700 (2) Å	µ = 1.01 mm^−^^1^
*c* = 32.3680 (7) Å	*T* = 295 K
*V* = 2945.21 (11) Å^3^	Chunk, yellow
*Z* = 4	0.36 × 0.20 × 0.18 mm

### Data collection

**Table:**

Rigaku R-AXIS RAPID/ZJUG diffractometer	6726 independent reflections
Radiation source: rolling anode	5515 reflections with *I* > 2σ(*I*)
graphite	*R*_int_ = 0.036
Detector resolution: 10.00 pixels mm^-1^	θ_max_ = 27.4°, θ_min_ = 3.0°
ω scans	*h* = −12→11
Absorption correction: multi-scan (*ABSCOR*; Higashi, 1995)	*k* = −12→12
*T*_min_ = 0.712, *T*_max_ = 0.839	*l* = −41→41
45235 measured reflections	

### Refinement

**Table:**

Refinement on *F*^2^	Secondary atom site location: difference Fourier map
Least-squares matrix: full	Hydrogen site location: inferred from neighbouring sites
*R*[*F*^2^ > 2σ(*F*^2^)] = 0.030	H-atom parameters constrained
*wR*(*F*^2^) = 0.077	*w* = 1/[σ^2^(*F*_o_^2^) + (0.0379*P*)^2^ + 0.4974*P*] where *P* = (*F*_o_^2^ + 2*F*_c_^2^)/3
*S* = 1.00	(Δ/σ)_max_ = 0.001
6726 reflections	Δρ_max_ = 0.37 e Å^−^^3^
361 parameters	Δρ_min_ = −0.23 e Å^−^^3^
30 restraints	Absolute structure: Flack (1983), 2915 Friedel pairs
Primary atom site location: structure-invariant direct methods	Flack parameter: −0.006 (14)

### Special details

**Table:**

Geometry. All e.s.d.'s (except the e.s.d. in the dihedral angle between two l.s. planes) are estimated using the full covariance matrix. The cell e.s.d.'s are taken into account individually in the estimation of e.s.d.'s in distances, angles and torsion angles; correlations between e.s.d.'s in cell parameters are only used when they are defined by crystal symmetry. An approximate (isotropic) treatment of cell e.s.d.'s is used for estimating e.s.d.'s involving l.s. planes.
Refinement. Refinement of *F*^2^ against ALL reflections. The weighted *R*-factor *wR* and goodness of fit *S* are based on *F*^2^, conventional *R*-factors *R* are based on *F*, with *F* set to zero for negative *F*^2^. The threshold expression of *F*^2^ > σ(*F*^2^) is used only for calculating *R*-factors(gt) *etc*. and is not relevant to the choice of reflections for refinement. *R*-factors based on *F*^2^ are statistically about twice as large as those based on *F*, and *R*- factors based on ALL data will be even larger.

### Fractional atomic coordinates and isotropic or equivalent isotropic displacement parameters (Å^2^)

**Table:**

	*x*	*y*	*z*	*U*_iso_*/*U*_eq_	
Fe1	0.43233 (3)	0.37036 (4)	0.323961 (10)	0.04776 (9)	
Fe2	0.94810 (3)	0.90157 (4)	0.321361 (10)	0.04747 (9)	
N2	0.9684 (2)	0.5571 (2)	0.41297 (5)	0.0485 (5)	
H102	0.9423	0.5072	0.3922	0.058*	
N1	0.7154 (2)	0.4016 (2)	0.40061 (6)	0.0503 (5)	
H101	0.6563	0.4670	0.3950	0.060*	
C3	0.6747 (2)	0.4068 (3)	0.47784 (7)	0.0452 (5)	
C2	0.8515 (2)	0.5632 (3)	0.44242 (6)	0.0409 (5)	
H2	0.7853	0.6356	0.4335	0.049*	
C1	0.7758 (2)	0.4207 (3)	0.44201 (6)	0.0425 (5)	
H1	0.8481	0.3486	0.4456	0.051*	
C9	0.9024 (2)	0.5980 (2)	0.48587 (6)	0.0401 (5)	
C8	0.7111 (3)	0.3287 (3)	0.51228 (8)	0.0598 (7)	
H8	0.7931	0.2757	0.5121	0.072*	
C14	1.0251 (3)	0.5382 (3)	0.50131 (7)	0.0500 (6)	
H14	1.0819	0.4847	0.4841	0.060*	
C15	0.6487 (3)	0.2668 (3)	0.39368 (8)	0.0548 (6)	
H15A	0.5734	0.2541	0.4135	0.066*	
H15B	0.7172	0.1930	0.3980	0.066*	
C26	1.0090 (2)	0.6932 (3)	0.39546 (7)	0.0471 (5)	
H26A	1.1022	0.6851	0.3835	0.057*	
H26B	1.0139	0.7613	0.4176	0.057*	
C10	0.8224 (3)	0.6798 (3)	0.51214 (8)	0.0521 (6)	
H10	0.7415	0.7229	0.5023	0.063*	
C31	0.8056 (2)	0.8517 (3)	0.36615 (8)	0.0549 (6)	
H31	0.7851	0.9072	0.3909	0.066*	
C16	0.5904 (3)	0.2557 (3)	0.35076 (7)	0.0508 (6)	
C27	0.9096 (2)	0.7459 (2)	0.36297 (6)	0.0422 (5)	
C13	1.0632 (3)	0.5577 (3)	0.54203 (8)	0.0601 (7)	
H13	1.1450	0.5166	0.5520	0.072*	
C12	0.9821 (3)	0.6369 (4)	0.56794 (8)	0.0678 (8)	
H12	1.0083	0.6489	0.5954	0.081*	
C28	0.9022 (3)	0.6931 (3)	0.32162 (7)	0.0543 (6)	
H28	0.9614	0.6189	0.3101	0.065*	
C11	0.8618 (3)	0.6985 (3)	0.55323 (9)	0.0652 (8)	
H11	0.8066	0.7527	0.5707	0.078*	
C4	0.5492 (3)	0.4811 (3)	0.47917 (8)	0.0598 (6)	
H4	0.5218	0.5326	0.4562	0.072*	
C6	0.5039 (4)	0.4050 (4)	0.54768 (10)	0.0793 (10)	
H6	0.4477	0.4053	0.5712	0.095*	
C20	0.4766 (3)	0.1674 (3)	0.33873 (9)	0.0592 (7)	
H20	0.4212	0.1076	0.3572	0.071*	
C17	0.6403 (3)	0.3242 (3)	0.31475 (8)	0.0614 (7)	
H17	0.7176	0.3918	0.3136	0.074*	
C29	0.7979 (3)	0.7670 (4)	0.30013 (9)	0.0698 (9)	
H29	0.7720	0.7531	0.2711	0.084*	
C32	1.1195 (4)	1.0080 (4)	0.34107 (11)	0.0839 (10)	
H32	1.1612	1.0017	0.3687	0.101*	
C5	0.4645 (3)	0.4805 (3)	0.51363 (10)	0.0715 (8)	
H5	0.3809	0.5311	0.5138	0.086*	
C30	0.7372 (3)	0.8646 (3)	0.32714 (10)	0.0702 (8)	
H30	0.6614	0.9302	0.3204	0.084*	
C24	0.2309 (3)	0.4341 (4)	0.31180 (11)	0.0801 (9)	
H24	0.1606	0.3797	0.2966	0.096*	
C18	0.5570 (3)	0.2791 (4)	0.28069 (8)	0.0732 (9)	
H18	0.5676	0.3094	0.2519	0.088*	
C19	0.4575 (3)	0.1810 (3)	0.29560 (9)	0.0710 (8)	
H19	0.3866	0.1324	0.2790	0.085*	
C7	0.6253 (4)	0.3293 (4)	0.54704 (9)	0.0785 (9)	
H7	0.6511	0.2775	0.5701	0.094*	
C23	0.3212 (4)	0.5237 (4)	0.29493 (11)	0.0834 (10)	
H23	0.3270	0.5443	0.2653	0.100*	
C25	0.2527 (4)	0.4278 (5)	0.35312 (12)	0.0930 (12)	
H25	0.1993	0.3707	0.3728	0.112*	
C35	1.0710 (4)	0.9670 (4)	0.27345 (10)	0.0883 (11)	
H35	1.0729	0.9273	0.2456	0.106*	
C33	1.0145 (4)	1.1026 (4)	0.32899 (12)	0.0847 (10)	
H33	0.9704	1.1742	0.3463	0.102*	
C34	0.9860 (4)	1.0758 (4)	0.28695 (11)	0.0871 (10)	
H34	0.9163	1.1252	0.2701	0.104*	
C21	0.3648 (5)	0.5201 (5)	0.36307 (12)	0.1072 (15)	
H21	0.4018	0.5418	0.3906	0.129*	
C36	1.1550 (3)	0.9248 (5)	0.30731 (13)	0.0906 (12)	
H36	1.2259	0.8504	0.3070	0.109*	
C22	0.4070 (4)	0.5806 (4)	0.32431 (17)	0.1047 (13)	
H22	0.4802	0.6512	0.3200	0.126*	

### Atomic displacement parameters (Å^2^)

**Table:**

	*U*^11^	*U*^22^	*U*^33^	*U*^12^	*U*^13^	*U*^23^
Fe1	0.05325 (19)	0.04715 (19)	0.04286 (16)	0.00223 (15)	0.00165 (15)	−0.00436 (15)
Fe2	0.04937 (17)	0.0520 (2)	0.04101 (16)	−0.00561 (15)	0.00362 (15)	0.00793 (15)
N2	0.0538 (11)	0.0516 (12)	0.0402 (10)	0.0026 (9)	0.0066 (9)	0.0041 (8)
N1	0.0605 (11)	0.0488 (12)	0.0416 (10)	−0.0036 (10)	−0.0095 (9)	0.0006 (9)
C3	0.0511 (12)	0.0417 (13)	0.0426 (11)	−0.0078 (10)	−0.0028 (9)	0.0007 (10)
C2	0.0410 (11)	0.0424 (13)	0.0395 (11)	0.0026 (9)	−0.0006 (9)	0.0067 (9)
C1	0.0450 (11)	0.0431 (13)	0.0393 (11)	0.0010 (10)	−0.0028 (9)	0.0029 (9)
C9	0.0372 (10)	0.0403 (12)	0.0427 (11)	−0.0056 (9)	0.0007 (9)	0.0022 (9)
C8	0.0799 (18)	0.0500 (16)	0.0495 (14)	0.0002 (14)	0.0017 (13)	0.0085 (12)
C14	0.0457 (13)	0.0517 (15)	0.0528 (14)	0.0016 (11)	−0.0024 (10)	0.0005 (11)
C15	0.0597 (15)	0.0459 (15)	0.0588 (14)	−0.0028 (11)	−0.0106 (12)	−0.0009 (12)
C26	0.0458 (12)	0.0544 (15)	0.0413 (11)	−0.0063 (10)	0.0005 (10)	0.0051 (10)
C10	0.0434 (12)	0.0559 (16)	0.0572 (14)	0.0022 (11)	−0.0015 (11)	−0.0082 (12)
C31	0.0498 (13)	0.0582 (17)	0.0565 (14)	−0.0026 (12)	0.0170 (11)	0.0018 (12)
C16	0.0503 (14)	0.0485 (14)	0.0535 (13)	0.0061 (11)	−0.0017 (11)	−0.0110 (11)
C27	0.0425 (12)	0.0466 (13)	0.0374 (10)	−0.0068 (10)	0.0061 (9)	0.0022 (9)
C13	0.0526 (14)	0.0753 (18)	0.0523 (13)	−0.0063 (14)	−0.0145 (13)	0.0087 (12)
C12	0.0673 (17)	0.090 (2)	0.0458 (14)	−0.0214 (16)	−0.0069 (12)	−0.0039 (14)
C28	0.0700 (15)	0.0517 (14)	0.0412 (11)	−0.0092 (12)	−0.0013 (12)	−0.0028 (12)
C11	0.0606 (17)	0.075 (2)	0.0603 (16)	−0.0106 (15)	0.0087 (14)	−0.0234 (15)
C4	0.0498 (14)	0.0714 (18)	0.0582 (14)	0.0008 (13)	−0.0009 (13)	0.0028 (12)
C6	0.091 (2)	0.076 (2)	0.0701 (19)	−0.0240 (19)	0.0350 (17)	−0.0107 (17)
C20	0.0592 (15)	0.0398 (14)	0.0786 (17)	0.0041 (11)	−0.0131 (13)	−0.0091 (12)
C17	0.0529 (14)	0.078 (2)	0.0527 (15)	0.0027 (13)	0.0100 (12)	−0.0141 (14)
C29	0.0742 (19)	0.084 (2)	0.0510 (15)	−0.0227 (17)	−0.0184 (14)	0.0103 (15)
C32	0.0736 (19)	0.105 (3)	0.0736 (19)	−0.0429 (19)	−0.0034 (16)	0.0102 (19)
C5	0.0549 (17)	0.080 (2)	0.0794 (18)	−0.0115 (15)	0.0153 (15)	−0.0085 (16)
C30	0.0423 (12)	0.083 (2)	0.086 (2)	−0.0008 (13)	−0.0033 (14)	0.0305 (19)
C24	0.0648 (17)	0.080 (2)	0.096 (3)	0.0132 (17)	−0.0069 (17)	0.0020 (19)
C18	0.0751 (19)	0.096 (2)	0.0483 (14)	0.0130 (19)	0.0027 (15)	−0.0240 (14)
C19	0.0755 (19)	0.0642 (19)	0.0734 (18)	0.0088 (17)	−0.0200 (16)	−0.0295 (15)
C7	0.117 (3)	0.067 (2)	0.0517 (15)	−0.013 (2)	0.0089 (17)	0.0135 (14)
C23	0.091 (2)	0.079 (3)	0.080 (2)	0.013 (2)	0.0009 (19)	0.0228 (19)
C25	0.096 (2)	0.097 (3)	0.086 (2)	0.045 (2)	0.039 (2)	0.023 (2)
C35	0.103 (3)	0.100 (3)	0.0622 (18)	−0.026 (2)	0.026 (2)	0.0171 (18)
C33	0.099 (2)	0.0587 (18)	0.096 (2)	−0.0257 (17)	0.0102 (19)	0.0013 (17)
C34	0.099 (2)	0.077 (2)	0.085 (2)	−0.020 (2)	−0.0014 (19)	0.0404 (19)
C21	0.129 (3)	0.111 (3)	0.081 (2)	0.075 (2)	−0.046 (2)	−0.057 (2)
C36	0.0541 (16)	0.097 (3)	0.121 (3)	−0.0101 (18)	0.0260 (18)	0.023 (2)
C22	0.088 (2)	0.0461 (18)	0.180 (4)	0.0015 (16)	−0.009 (3)	−0.010 (2)

### Geometric parameters (Å, °)

**Table:**

Fe1—C21	2.017 (3)	C31—H31	0.9800
Fe1—C22	2.027 (3)	C16—C17	1.419 (4)
Fe1—C25	2.028 (3)	C16—C20	1.427 (4)
Fe1—C18	2.032 (3)	C27—C28	1.432 (3)
Fe1—C23	2.038 (3)	C13—C12	1.368 (4)
Fe1—C20	2.044 (3)	C13—H13	0.9300
Fe1—C19	2.045 (3)	C12—C11	1.371 (4)
Fe1—C24	2.048 (3)	C12—H12	0.9300
Fe1—C17	2.048 (3)	C28—C29	1.403 (4)
Fe1—C16	2.053 (3)	C28—H28	0.9800
Fe2—C32	2.025 (3)	C11—H11	0.9300
Fe2—C36	2.031 (3)	C4—C5	1.376 (4)
Fe2—C34	2.037 (3)	C4—H4	0.9300
Fe2—C33	2.040 (3)	C6—C7	1.363 (5)
Fe2—C35	2.040 (3)	C6—C5	1.370 (5)
Fe2—C31	2.041 (2)	C6—H6	0.9300
Fe2—C27	2.041 (2)	C20—C19	1.414 (4)
Fe2—C28	2.043 (3)	C20—H20	0.9800
Fe2—C29	2.042 (3)	C17—C18	1.425 (4)
Fe2—C30	2.045 (3)	C17—H17	0.9800
N2—C2	1.466 (3)	C29—C30	1.403 (4)
N2—C26	1.472 (3)	C29—H29	0.9800
N2—H102	0.8600	C32—C36	1.394 (5)
N1—C15	1.455 (3)	C32—C33	1.403 (5)
N1—C1	1.469 (3)	C32—H32	0.9800
N1—H101	0.8599	C5—H5	0.9300
C3—C8	1.386 (3)	C30—H30	0.9800
C3—C4	1.389 (3)	C24—C23	1.331 (5)
C3—C1	1.513 (3)	C24—C25	1.355 (5)
C2—C9	1.524 (3)	C24—H24	0.9800
C2—C1	1.542 (3)	C18—C19	1.418 (4)
C2—H2	0.9800	C18—H18	0.9800
C1—H1	0.9800	C19—H19	0.9800
C9—C10	1.384 (3)	C7—H7	0.9300
C9—C14	1.393 (3)	C23—C22	1.366 (5)
C8—C7	1.389 (4)	C23—H23	0.9800
C8—H8	0.9300	C25—C21	1.422 (6)
C14—C13	1.379 (3)	C25—H25	0.9800
C14—H14	0.9300	C35—C34	1.388 (5)
C15—C16	1.500 (3)	C35—C36	1.415 (5)
C15—H15A	0.9700	C35—H35	0.9800
C15—H15B	0.9700	C33—C34	1.411 (5)
C26—C27	1.501 (3)	C33—H33	0.9800
C26—H26A	0.9700	C34—H34	0.9800
C26—H26B	0.9700	C21—C22	1.439 (6)
C10—C11	1.393 (4)	C21—H21	0.9800
C10—H10	0.9300	C36—H36	0.9800
C31—C27	1.419 (3)	C22—H22	0.9800
C31—C30	1.426 (4)		
			
C21—Fe1—C22	41.69 (17)	C27—C31—H31	125.8
C21—Fe1—C25	41.16 (17)	C30—C31—H31	125.8
C22—Fe1—C25	68.18 (17)	Fe2—C31—H31	125.8
C21—Fe1—C18	157.5 (2)	C17—C16—C20	107.6 (2)
C22—Fe1—C18	119.99 (18)	C17—C16—C15	127.2 (2)
C25—Fe1—C18	158.24 (17)	C20—C16—C15	125.1 (2)
C21—Fe1—C23	67.22 (15)	C17—C16—Fe1	69.57 (15)
C22—Fe1—C23	39.29 (15)	C20—C16—Fe1	69.28 (14)
C25—Fe1—C23	65.34 (14)	C15—C16—Fe1	128.65 (18)
C18—Fe1—C23	107.09 (14)	C31—C27—C28	106.6 (2)
C21—Fe1—C20	126.43 (17)	C31—C27—C26	128.9 (2)
C22—Fe1—C20	165.16 (17)	C28—C27—C26	124.6 (2)
C25—Fe1—C20	108.78 (14)	C31—C27—Fe2	69.63 (13)
C18—Fe1—C20	68.46 (13)	C28—C27—Fe2	69.51 (13)
C23—Fe1—C20	154.00 (14)	C26—C27—Fe2	126.48 (16)
C21—Fe1—C19	161.5 (2)	C12—C13—C14	120.8 (3)
C22—Fe1—C19	153.65 (19)	C12—C13—H13	119.6
C25—Fe1—C19	123.20 (17)	C14—C13—H13	119.6
C18—Fe1—C19	40.69 (13)	C13—C12—C11	119.7 (2)
C23—Fe1—C19	119.45 (14)	C13—C12—H12	120.2
C20—Fe1—C19	40.45 (11)	C11—C12—H12	120.2
C21—Fe1—C24	67.14 (15)	C29—C28—C27	108.7 (3)
C22—Fe1—C24	66.07 (16)	C29—C28—Fe2	69.90 (16)
C25—Fe1—C24	38.83 (14)	C27—C28—Fe2	69.43 (14)
C18—Fe1—C24	122.74 (13)	C29—C28—H28	125.7
C23—Fe1—C24	38.02 (14)	C27—C28—H28	125.7
C20—Fe1—C24	121.35 (14)	Fe2—C28—H28	125.7
C19—Fe1—C24	106.67 (14)	C12—C11—C10	120.0 (3)
C21—Fe1—C17	123.49 (16)	C12—C11—H11	120.0
C22—Fe1—C17	109.24 (15)	C10—C11—H11	120.0
C25—Fe1—C17	160.00 (15)	C5—C4—C3	121.7 (3)
C18—Fe1—C17	40.88 (11)	C5—C4—H4	119.1
C23—Fe1—C17	126.06 (14)	C3—C4—H4	119.1
C20—Fe1—C17	68.31 (12)	C7—C6—C5	120.0 (3)
C19—Fe1—C17	68.33 (13)	C7—C6—H6	120.0
C24—Fe1—C17	159.91 (13)	C5—C6—H6	120.0
C21—Fe1—C16	110.33 (12)	C19—C20—C16	108.2 (3)
C22—Fe1—C16	127.96 (15)	C19—C20—Fe1	69.82 (17)
C25—Fe1—C16	124.39 (13)	C16—C20—Fe1	69.94 (15)
C18—Fe1—C16	68.58 (11)	C19—C20—H20	125.9
C23—Fe1—C16	163.57 (14)	C16—C20—H20	125.9
C20—Fe1—C16	40.77 (10)	Fe1—C20—H20	125.9
C19—Fe1—C16	68.32 (11)	C16—C17—C18	108.0 (3)
C24—Fe1—C16	157.64 (13)	C16—C17—Fe1	69.94 (14)
C17—Fe1—C16	40.49 (11)	C18—C17—Fe1	68.95 (15)
C32—Fe2—C36	40.19 (15)	C16—C17—H17	126.0
C32—Fe2—C34	67.56 (15)	C18—C17—H17	126.0
C36—Fe2—C34	67.48 (16)	Fe1—C17—H17	126.0
C32—Fe2—C33	40.38 (15)	C30—C29—C28	108.5 (2)
C36—Fe2—C33	67.92 (17)	C30—C29—Fe2	70.03 (17)
C34—Fe2—C33	40.50 (14)	C28—C29—Fe2	69.93 (15)
C32—Fe2—C35	67.93 (14)	C30—C29—H29	125.7
C36—Fe2—C35	40.68 (14)	C28—C29—H29	125.7
C34—Fe2—C35	39.82 (14)	Fe2—C29—H29	125.7
C33—Fe2—C35	67.99 (15)	C36—C32—C33	108.8 (3)
C32—Fe2—C31	115.35 (13)	C36—C32—Fe2	70.16 (19)
C36—Fe2—C31	145.84 (14)	C33—C32—Fe2	70.39 (19)
C34—Fe2—C31	134.21 (15)	C36—C32—H32	125.6
C33—Fe2—C31	109.89 (13)	C33—C32—H32	125.6
C35—Fe2—C31	172.72 (14)	Fe2—C32—H32	125.6
C32—Fe2—C27	107.67 (12)	C6—C5—C4	119.6 (3)
C36—Fe2—C27	113.64 (14)	C6—C5—H5	120.2
C34—Fe2—C27	171.77 (14)	C4—C5—H5	120.2
C33—Fe2—C27	131.60 (12)	C29—C30—C31	107.8 (2)
C35—Fe2—C27	145.91 (14)	C29—C30—Fe2	69.81 (16)
C31—Fe2—C27	40.68 (10)	C31—C30—Fe2	69.40 (13)
C32—Fe2—C28	131.46 (15)	C29—C30—H30	126.1
C36—Fe2—C28	108.35 (15)	C31—C30—H30	126.1
C34—Fe2—C28	147.09 (14)	Fe2—C30—H30	126.1
C33—Fe2—C28	170.75 (14)	C23—C24—C25	109.6 (4)
C35—Fe2—C28	115.18 (14)	C23—C24—Fe1	70.6 (2)
C31—Fe2—C28	68.07 (11)	C25—C24—Fe1	69.8 (2)
C27—Fe2—C28	41.06 (9)	C23—C24—H24	125.2
C32—Fe2—C29	170.43 (17)	C25—C24—H24	125.2
C36—Fe2—C29	132.14 (17)	Fe1—C24—H24	125.2
C34—Fe2—C29	117.11 (13)	C19—C18—C17	108.0 (3)
C33—Fe2—C29	148.42 (15)	C19—C18—Fe1	70.17 (16)
C35—Fe2—C29	109.77 (14)	C17—C18—Fe1	70.18 (14)
C31—Fe2—C29	68.11 (12)	C19—C18—H18	126.0
C27—Fe2—C29	68.68 (10)	C17—C18—H18	126.0
C28—Fe2—C29	40.17 (11)	Fe1—C18—H18	126.0
C32—Fe2—C30	147.94 (16)	C20—C19—C18	108.2 (3)
C36—Fe2—C30	171.54 (16)	C20—C19—Fe1	69.73 (15)
C34—Fe2—C30	111.41 (14)	C18—C19—Fe1	69.14 (16)
C33—Fe2—C30	117.11 (15)	C20—C19—H19	125.9
C35—Fe2—C30	133.17 (14)	C18—C19—H19	125.9
C31—Fe2—C30	40.85 (11)	Fe1—C19—H19	125.9
C27—Fe2—C30	68.75 (10)	C6—C7—C8	120.8 (3)
C28—Fe2—C30	67.72 (12)	C6—C7—H7	119.6
C29—Fe2—C30	40.16 (13)	C8—C7—H7	119.6
C2—N2—C26	114.44 (19)	C24—C23—C22	110.9 (4)
C2—N2—H102	108.1	C24—C23—Fe1	71.4 (2)
C26—N2—H102	105.5	C22—C23—Fe1	69.9 (2)
C15—N1—C1	114.88 (19)	C24—C23—H23	124.6
C15—N1—H101	109.1	C22—C23—H23	124.6
C1—N1—H101	111.0	Fe1—C23—H23	124.6
C8—C3—C4	117.7 (2)	C24—C25—C21	108.1 (4)
C8—C3—C1	120.3 (2)	C24—C25—Fe1	71.39 (19)
C4—C3—C1	121.6 (2)	C21—C25—Fe1	69.0 (2)
N2—C2—C9	111.58 (17)	C24—C25—H25	126.0
N2—C2—C1	108.22 (19)	C21—C25—H25	126.0
C9—C2—C1	110.45 (17)	Fe1—C25—H25	126.0
N2—C2—H2	108.8	C34—C35—C36	107.4 (3)
C9—C2—H2	108.8	C34—C35—Fe2	69.98 (18)
C1—C2—H2	108.8	C36—C35—Fe2	69.32 (17)
N1—C1—C3	116.08 (19)	C34—C35—H35	126.3
N1—C1—C2	107.45 (17)	C36—C35—H35	126.3
C3—C1—C2	111.57 (19)	Fe2—C35—H35	126.3
N1—C1—H1	107.1	C32—C33—C34	106.8 (3)
C3—C1—H1	107.1	C32—C33—Fe2	69.23 (19)
C2—C1—H1	107.1	C34—C33—Fe2	69.65 (19)
C10—C9—C14	118.2 (2)	C32—C33—H33	126.6
C10—C9—C2	121.1 (2)	C34—C33—H33	126.6
C14—C9—C2	120.5 (2)	Fe2—C33—H33	126.6
C3—C8—C7	120.2 (3)	C35—C34—C33	109.1 (3)
C3—C8—H8	119.9	C35—C34—Fe2	70.20 (19)
C7—C8—H8	119.9	C33—C34—Fe2	69.84 (17)
C13—C14—C9	120.5 (2)	C35—C34—H34	125.4
C13—C14—H14	119.8	C33—C34—H34	125.4
C9—C14—H14	119.8	Fe2—C34—H34	125.4
N1—C15—C16	111.5 (2)	C25—C21—C22	105.2 (3)
N1—C15—H15A	109.3	C25—C21—Fe1	69.81 (18)
C16—C15—H15A	109.3	C22—C21—Fe1	69.51 (19)
N1—C15—H15B	109.3	C25—C21—H21	127.4
C16—C15—H15B	109.3	C22—C21—H21	127.4
H15A—C15—H15B	108.0	Fe1—C21—H21	127.4
N2—C26—C27	113.71 (19)	C32—C36—C35	107.9 (4)
N2—C26—H26A	108.8	C32—C36—Fe2	69.65 (18)
C27—C26—H26A	108.8	C35—C36—Fe2	70.00 (18)
N2—C26—H26B	108.8	C32—C36—H36	126.1
C27—C26—H26B	108.8	C35—C36—H36	126.1
H26A—C26—H26B	107.7	Fe2—C36—H36	126.1
C9—C10—C11	120.8 (2)	C23—C22—C21	106.3 (3)
C9—C10—H10	119.6	C23—C22—Fe1	70.8 (2)
C11—C10—H10	119.6	C21—C22—Fe1	68.8 (2)
C27—C31—C30	108.4 (2)	C23—C22—H22	126.9
C27—C31—Fe2	69.69 (12)	C21—C22—H22	126.9
C30—C31—Fe2	69.75 (14)	Fe1—C22—H22	126.9
			
C26—N2—C2—C9	−85.0 (2)	C27—Fe2—C30—C31	37.41 (16)
C26—N2—C2—C1	153.26 (18)	C28—Fe2—C30—C31	81.77 (17)
C15—N1—C1—C3	−58.8 (3)	C29—Fe2—C30—C31	119.1 (3)
C15—N1—C1—C2	175.5 (2)	C21—Fe1—C24—C23	81.8 (3)
C8—C3—C1—N1	134.4 (2)	C22—Fe1—C24—C23	36.2 (3)
C4—C3—C1—N1	−51.6 (3)	C25—Fe1—C24—C23	120.6 (4)
C8—C3—C1—C2	−102.1 (3)	C18—Fe1—C24—C23	−75.2 (3)
C4—C3—C1—C2	71.9 (3)	C20—Fe1—C24—C23	−158.4 (2)
N2—C2—C1—N1	−64.2 (2)	C19—Fe1—C24—C23	−116.8 (3)
C9—C2—C1—N1	173.44 (18)	C17—Fe1—C24—C23	−44.4 (5)
N2—C2—C1—C3	167.55 (17)	C16—Fe1—C24—C23	169.8 (3)
C9—C2—C1—C3	45.1 (2)	C21—Fe1—C24—C25	−38.8 (3)
N2—C2—C9—C10	144.6 (2)	C22—Fe1—C24—C25	−84.4 (3)
C1—C2—C9—C10	−95.0 (3)	C18—Fe1—C24—C25	164.2 (3)
N2—C2—C9—C14	−40.9 (3)	C23—Fe1—C24—C25	−120.6 (4)
C1—C2—C9—C14	79.5 (2)	C20—Fe1—C24—C25	81.0 (3)
C4—C3—C8—C7	−1.7 (4)	C19—Fe1—C24—C25	122.6 (3)
C1—C3—C8—C7	172.5 (3)	C17—Fe1—C24—C25	−165.0 (4)
C10—C9—C14—C13	1.6 (4)	C16—Fe1—C24—C25	49.3 (5)
C2—C9—C14—C13	−173.0 (2)	C16—C17—C18—C19	−1.1 (3)
C1—N1—C15—C16	−179.8 (2)	Fe1—C17—C18—C19	−60.3 (2)
C2—N2—C26—C27	−76.0 (2)	C16—C17—C18—Fe1	59.21 (19)
C14—C9—C10—C11	−1.8 (4)	C21—Fe1—C18—C19	172.9 (3)
C2—C9—C10—C11	172.8 (2)	C22—Fe1—C18—C19	−156.3 (2)
C32—Fe2—C31—C27	−87.9 (2)	C25—Fe1—C18—C19	−49.6 (5)
C36—Fe2—C31—C27	−51.8 (3)	C23—Fe1—C18—C19	−115.6 (2)
C34—Fe2—C31—C27	−170.57 (18)	C20—Fe1—C18—C19	37.27 (18)
C33—Fe2—C31—C27	−131.48 (17)	C24—Fe1—C18—C19	−77.0 (2)
C28—Fe2—C31—C27	38.86 (14)	C17—Fe1—C18—C19	118.6 (3)
C29—Fe2—C31—C27	82.32 (17)	C16—Fe1—C18—C19	81.24 (19)
C30—Fe2—C31—C27	119.7 (2)	C21—Fe1—C18—C17	54.3 (4)
C32—Fe2—C31—C30	152.4 (2)	C22—Fe1—C18—C17	85.1 (2)
C36—Fe2—C31—C30	−171.5 (3)	C25—Fe1—C18—C17	−168.2 (4)
C34—Fe2—C31—C30	69.7 (3)	C23—Fe1—C18—C17	125.9 (2)
C33—Fe2—C31—C30	108.8 (2)	C20—Fe1—C18—C17	−81.3 (2)
C27—Fe2—C31—C30	−119.7 (2)	C19—Fe1—C18—C17	−118.6 (3)
C28—Fe2—C31—C30	−80.83 (19)	C24—Fe1—C18—C17	164.4 (2)
C29—Fe2—C31—C30	−37.38 (19)	C16—Fe1—C18—C17	−37.34 (18)
N1—C15—C16—C17	28.7 (4)	C16—C20—C19—C18	−1.0 (3)
N1—C15—C16—C20	−154.4 (2)	Fe1—C20—C19—C18	58.6 (2)
N1—C15—C16—Fe1	−64.0 (3)	C16—C20—C19—Fe1	−59.64 (18)
C21—Fe1—C16—C17	−118.2 (2)	C17—C18—C19—C20	1.3 (3)
C22—Fe1—C16—C17	−74.3 (3)	Fe1—C18—C19—C20	−59.0 (2)
C25—Fe1—C16—C17	−162.2 (2)	C17—C18—C19—Fe1	60.3 (2)
C18—Fe1—C16—C17	37.69 (18)	C21—Fe1—C19—C20	−51.6 (5)
C23—Fe1—C16—C17	−39.9 (5)	C22—Fe1—C19—C20	171.4 (3)
C20—Fe1—C16—C17	119.1 (2)	C25—Fe1—C19—C20	−80.0 (2)
C19—Fe1—C16—C17	81.59 (18)	C18—Fe1—C19—C20	119.8 (3)
C24—Fe1—C16—C17	162.7 (3)	C23—Fe1—C19—C20	−158.24 (19)
C21—Fe1—C16—C20	122.7 (2)	C24—Fe1—C19—C20	−119.1 (2)
C22—Fe1—C16—C20	166.6 (2)	C17—Fe1—C19—C20	81.55 (19)
C25—Fe1—C16—C20	78.7 (2)	C16—Fe1—C19—C20	37.83 (17)
C18—Fe1—C16—C20	−81.43 (18)	C21—Fe1—C19—C18	−171.4 (4)
C23—Fe1—C16—C20	−159.0 (4)	C22—Fe1—C19—C18	51.6 (4)
C19—Fe1—C16—C20	−37.54 (18)	C25—Fe1—C19—C18	160.29 (19)
C24—Fe1—C16—C20	43.5 (4)	C23—Fe1—C19—C18	82.0 (2)
C17—Fe1—C16—C20	−119.1 (2)	C20—Fe1—C19—C18	−119.8 (3)
C21—Fe1—C16—C15	3.7 (3)	C24—Fe1—C19—C18	121.18 (19)
C22—Fe1—C16—C15	47.5 (3)	C17—Fe1—C19—C18	−38.20 (17)
C25—Fe1—C16—C15	−40.3 (3)	C16—Fe1—C19—C18	−81.92 (19)
C18—Fe1—C16—C15	159.6 (3)	C5—C6—C7—C8	0.6 (5)
C23—Fe1—C16—C15	81.9 (5)	C3—C8—C7—C6	0.8 (5)
C20—Fe1—C16—C15	−119.0 (3)	C25—C24—C23—C22	0.1 (4)
C19—Fe1—C16—C15	−156.5 (3)	Fe1—C24—C23—C22	−58.9 (3)
C24—Fe1—C16—C15	−75.5 (4)	C25—C24—C23—Fe1	59.0 (3)
C17—Fe1—C16—C15	121.9 (3)	C21—Fe1—C23—C24	−81.5 (3)
C30—C31—C27—C28	−0.8 (3)	C22—Fe1—C23—C24	−121.6 (4)
Fe2—C31—C27—C28	−60.00 (16)	C25—Fe1—C23—C24	−36.4 (2)
C30—C31—C27—C26	−179.8 (2)	C18—Fe1—C23—C24	121.7 (2)
Fe2—C31—C27—C26	121.0 (2)	C20—Fe1—C23—C24	45.9 (4)
C30—C31—C27—Fe2	59.19 (17)	C19—Fe1—C23—C24	79.2 (3)
N2—C26—C27—C31	103.7 (3)	C17—Fe1—C23—C24	162.7 (2)
N2—C26—C27—C28	−75.1 (3)	C16—Fe1—C23—C24	−166.3 (4)
N2—C26—C27—Fe2	−164.03 (15)	C21—Fe1—C23—C22	40.0 (3)
C32—Fe2—C27—C31	108.58 (19)	C25—Fe1—C23—C22	85.1 (3)
C36—Fe2—C27—C31	151.2 (2)	C18—Fe1—C23—C22	−116.7 (3)
C33—Fe2—C27—C31	70.4 (2)	C20—Fe1—C23—C22	167.5 (3)
C35—Fe2—C27—C31	−174.9 (2)	C19—Fe1—C23—C22	−159.2 (3)
C28—Fe2—C27—C31	−117.6 (2)	C24—Fe1—C23—C22	121.6 (4)
C29—Fe2—C27—C31	−80.80 (18)	C17—Fe1—C23—C22	−75.7 (3)
C30—Fe2—C27—C31	−37.56 (17)	C16—Fe1—C23—C22	−44.7 (6)
C32—Fe2—C27—C28	−133.82 (18)	C23—C24—C25—C21	0.0 (4)
C36—Fe2—C27—C28	−91.2 (2)	Fe1—C24—C25—C21	59.5 (2)
C33—Fe2—C27—C28	−172.0 (2)	C23—C24—C25—Fe1	−59.5 (3)
C35—Fe2—C27—C28	−57.3 (3)	C21—Fe1—C25—C24	118.7 (3)
C31—Fe2—C27—C28	117.6 (2)	C22—Fe1—C25—C24	78.5 (3)
C29—Fe2—C27—C28	36.80 (17)	C18—Fe1—C25—C24	−38.2 (5)
C30—Fe2—C27—C28	80.04 (18)	C23—Fe1—C25—C24	35.7 (2)
C32—Fe2—C27—C26	−15.3 (2)	C20—Fe1—C25—C24	−117.0 (2)
C36—Fe2—C27—C26	27.3 (3)	C19—Fe1—C25—C24	−74.6 (3)
C33—Fe2—C27—C26	−53.5 (3)	C17—Fe1—C25—C24	164.9 (4)
C35—Fe2—C27—C26	61.2 (3)	C16—Fe1—C25—C24	−159.6 (2)
C31—Fe2—C27—C26	−123.9 (3)	C22—Fe1—C25—C21	−40.2 (2)
C28—Fe2—C27—C26	118.5 (3)	C18—Fe1—C25—C21	−156.9 (4)
C29—Fe2—C27—C26	155.3 (2)	C23—Fe1—C25—C21	−83.0 (2)
C30—Fe2—C27—C26	−161.5 (2)	C20—Fe1—C25—C21	124.3 (2)
C9—C14—C13—C12	−0.5 (4)	C19—Fe1—C25—C21	166.8 (2)
C14—C13—C12—C11	−0.4 (4)	C24—Fe1—C25—C21	−118.7 (3)
C31—C27—C28—C29	1.0 (3)	C17—Fe1—C25—C21	46.3 (5)
C26—C27—C28—C29	−179.9 (2)	C16—Fe1—C25—C21	81.8 (3)
Fe2—C27—C28—C29	−59.04 (18)	C32—Fe2—C35—C34	−81.0 (3)
C31—C27—C28—Fe2	60.08 (16)	C36—Fe2—C35—C34	−118.5 (4)
C26—C27—C28—Fe2	−120.9 (2)	C33—Fe2—C35—C34	−37.3 (2)
C32—Fe2—C28—C29	−173.3 (2)	C27—Fe2—C35—C34	−170.1 (2)
C36—Fe2—C28—C29	−134.7 (2)	C28—Fe2—C35—C34	152.2 (2)
C34—Fe2—C28—C29	−58.0 (3)	C29—Fe2—C35—C34	109.0 (2)
C35—Fe2—C28—C29	−91.3 (2)	C30—Fe2—C35—C34	69.9 (3)
C31—Fe2—C28—C29	81.60 (18)	C32—Fe2—C35—C36	37.6 (2)
C27—Fe2—C28—C29	120.1 (2)	C34—Fe2—C35—C36	118.5 (4)
C30—Fe2—C28—C29	37.35 (17)	C33—Fe2—C35—C36	81.3 (3)
C32—Fe2—C28—C27	66.6 (2)	C27—Fe2—C35—C36	−51.6 (3)
C36—Fe2—C28—C27	105.22 (19)	C28—Fe2—C35—C36	−89.2 (3)
C34—Fe2—C28—C27	−178.1 (2)	C29—Fe2—C35—C36	−132.5 (3)
C35—Fe2—C28—C27	148.59 (17)	C30—Fe2—C35—C36	−171.6 (2)
C31—Fe2—C28—C27	−38.51 (14)	C36—C32—C33—C34	0.0 (4)
C29—Fe2—C28—C27	−120.1 (2)	Fe2—C32—C33—C34	−59.8 (2)
C30—Fe2—C28—C27	−82.77 (16)	C36—C32—C33—Fe2	59.9 (2)
C13—C12—C11—C10	0.3 (5)	C36—Fe2—C33—C32	−37.3 (2)
C9—C10—C11—C12	0.8 (4)	C34—Fe2—C33—C32	−118.0 (3)
C8—C3—C4—C5	1.4 (4)	C35—Fe2—C33—C32	−81.4 (2)
C1—C3—C4—C5	−172.8 (3)	C31—Fe2—C33—C32	106.1 (2)
C17—C16—C20—C19	0.4 (3)	C27—Fe2—C33—C32	65.3 (3)
C15—C16—C20—C19	−177.0 (2)	C29—Fe2—C33—C32	−173.6 (2)
Fe1—C16—C20—C19	59.57 (19)	C30—Fe2—C33—C32	150.2 (2)
C17—C16—C20—Fe1	−59.21 (19)	C32—Fe2—C33—C34	118.0 (3)
C15—C16—C20—Fe1	123.4 (2)	C36—Fe2—C33—C34	80.7 (2)
C21—Fe1—C20—C19	162.0 (2)	C35—Fe2—C33—C34	36.6 (2)
C22—Fe1—C20—C19	−164.9 (5)	C31—Fe2—C33—C34	−135.9 (2)
C25—Fe1—C20—C19	119.5 (2)	C27—Fe2—C33—C34	−176.7 (2)
C18—Fe1—C20—C19	−37.48 (18)	C29—Fe2—C33—C34	−55.6 (4)
C23—Fe1—C20—C19	47.4 (4)	C30—Fe2—C33—C34	−91.8 (2)
C24—Fe1—C20—C19	78.6 (2)	C36—C35—C34—C33	−0.3 (4)
C17—Fe1—C20—C19	−81.61 (19)	Fe2—C35—C34—C33	59.2 (2)
C16—Fe1—C20—C19	−119.2 (3)	C36—C35—C34—Fe2	−59.5 (2)
C21—Fe1—C20—C16	−78.8 (2)	C32—C33—C34—C35	0.2 (4)
C22—Fe1—C20—C16	−45.7 (6)	Fe2—C33—C34—C35	−59.4 (2)
C25—Fe1—C20—C16	−121.3 (2)	C32—C33—C34—Fe2	59.6 (2)
C18—Fe1—C20—C16	81.75 (17)	C32—Fe2—C34—C35	82.0 (3)
C23—Fe1—C20—C16	166.7 (3)	C36—Fe2—C34—C35	38.3 (2)
C19—Fe1—C20—C16	119.2 (3)	C33—Fe2—C34—C35	120.2 (3)
C24—Fe1—C20—C16	−162.13 (17)	C31—Fe2—C34—C35	−173.9 (2)
C17—Fe1—C20—C16	37.62 (15)	C28—Fe2—C34—C35	−50.9 (4)
C20—C16—C17—C18	0.4 (3)	C29—Fe2—C34—C35	−88.8 (3)
C15—C16—C17—C18	177.8 (3)	C30—Fe2—C34—C35	−132.6 (2)
Fe1—C16—C17—C18	−58.6 (2)	C32—Fe2—C34—C33	−38.2 (2)
C20—C16—C17—Fe1	59.03 (18)	C36—Fe2—C34—C33	−81.9 (3)
C15—C16—C17—Fe1	−123.6 (3)	C35—Fe2—C34—C33	−120.2 (3)
C21—Fe1—C17—C16	82.3 (2)	C31—Fe2—C34—C33	65.9 (3)
C22—Fe1—C17—C16	126.5 (2)	C28—Fe2—C34—C33	−171.1 (2)
C25—Fe1—C17—C16	47.6 (5)	C29—Fe2—C34—C33	151.0 (2)
C18—Fe1—C17—C16	−119.6 (3)	C30—Fe2—C34—C33	107.1 (2)
C23—Fe1—C17—C16	167.03 (19)	C24—C25—C21—C22	−0.1 (4)
C20—Fe1—C17—C16	−37.88 (15)	Fe1—C25—C21—C22	60.9 (2)
C19—Fe1—C17—C16	−81.56 (18)	C24—C25—C21—Fe1	−61.0 (2)
C24—Fe1—C17—C16	−160.7 (4)	C22—Fe1—C21—C25	115.8 (3)
C21—Fe1—C17—C18	−158.1 (3)	C18—Fe1—C21—C25	157.7 (3)
C22—Fe1—C17—C18	−113.9 (3)	C23—Fe1—C21—C25	78.0 (2)
C25—Fe1—C17—C18	167.1 (4)	C20—Fe1—C21—C25	−76.3 (2)
C23—Fe1—C17—C18	−73.4 (3)	C19—Fe1—C21—C25	−37.1 (5)
C20—Fe1—C17—C18	81.7 (2)	C24—Fe1—C21—C25	36.7 (2)
C19—Fe1—C17—C18	38.0 (2)	C17—Fe1—C21—C25	−162.8 (2)
C24—Fe1—C17—C18	−41.2 (5)	C16—Fe1—C21—C25	−119.4 (2)
C16—Fe1—C17—C18	119.6 (3)	C25—Fe1—C21—C22	−115.8 (3)
C27—C28—C29—C30	−0.9 (3)	C18—Fe1—C21—C22	41.9 (4)
Fe2—C28—C29—C30	−59.6 (2)	C23—Fe1—C21—C22	−37.8 (2)
C27—C28—C29—Fe2	58.75 (17)	C20—Fe1—C21—C22	167.9 (2)
C36—Fe2—C29—C30	−174.9 (2)	C19—Fe1—C21—C22	−152.9 (4)
C34—Fe2—C29—C30	−91.7 (2)	C24—Fe1—C21—C22	−79.1 (2)
C33—Fe2—C29—C30	−54.7 (3)	C17—Fe1—C21—C22	81.4 (2)
C35—Fe2—C29—C30	−134.5 (2)	C16—Fe1—C21—C22	124.8 (2)
C31—Fe2—C29—C30	38.01 (17)	C33—C32—C36—C35	−0.2 (4)
C27—Fe2—C29—C30	81.91 (17)	Fe2—C32—C36—C35	59.8 (2)
C28—Fe2—C29—C30	119.5 (2)	C33—C32—C36—Fe2	−60.0 (2)
C36—Fe2—C29—C28	65.6 (2)	C34—C35—C36—C32	0.3 (4)
C34—Fe2—C29—C28	148.84 (19)	Fe2—C35—C36—C32	−59.6 (2)
C33—Fe2—C29—C28	−174.2 (2)	C34—C35—C36—Fe2	59.9 (2)
C35—Fe2—C29—C28	106.0 (2)	C34—Fe2—C36—C32	81.4 (3)
C31—Fe2—C29—C28	−81.48 (17)	C33—Fe2—C36—C32	37.5 (2)
C27—Fe2—C29—C28	−37.58 (15)	C35—Fe2—C36—C32	118.9 (4)
C30—Fe2—C29—C28	−119.5 (2)	C31—Fe2—C36—C32	−55.7 (4)
C34—Fe2—C32—C36	−81.2 (3)	C27—Fe2—C36—C32	−89.7 (2)
C33—Fe2—C32—C36	−119.5 (3)	C28—Fe2—C36—C32	−133.5 (2)
C35—Fe2—C32—C36	−38.0 (2)	C29—Fe2—C36—C32	−171.7 (2)
C31—Fe2—C32—C36	149.1 (2)	C32—Fe2—C36—C35	−118.9 (4)
C27—Fe2—C32—C36	106.0 (2)	C34—Fe2—C36—C35	−37.5 (2)
C28—Fe2—C32—C36	66.7 (3)	C33—Fe2—C36—C35	−81.5 (3)
C30—Fe2—C32—C36	−176.0 (2)	C31—Fe2—C36—C35	−174.7 (2)
C36—Fe2—C32—C33	119.5 (3)	C27—Fe2—C36—C35	151.3 (2)
C34—Fe2—C32—C33	38.3 (2)	C28—Fe2—C36—C35	107.6 (2)
C35—Fe2—C32—C33	81.5 (2)	C29—Fe2—C36—C35	69.3 (3)
C31—Fe2—C32—C33	−91.4 (2)	C24—C23—C22—C21	−0.2 (4)
C27—Fe2—C32—C33	−134.5 (2)	Fe1—C23—C22—C21	−60.0 (2)
C28—Fe2—C32—C33	−173.7 (2)	C24—C23—C22—Fe1	59.8 (3)
C30—Fe2—C32—C33	−56.5 (3)	C25—C21—C22—C23	0.2 (4)
C7—C6—C5—C4	−1.0 (5)	Fe1—C21—C22—C23	61.3 (3)
C3—C4—C5—C6	0.0 (5)	C25—C21—C22—Fe1	−61.1 (2)
C28—C29—C30—C31	0.4 (3)	C21—Fe1—C22—C23	−116.9 (3)
Fe2—C29—C30—C31	−59.20 (19)	C25—Fe1—C22—C23	−77.3 (2)
C28—C29—C30—Fe2	59.56 (19)	C18—Fe1—C22—C23	80.2 (3)
C27—C31—C30—C29	0.3 (3)	C20—Fe1—C22—C23	−158.3 (5)
Fe2—C31—C30—C29	59.5 (2)	C19—Fe1—C22—C23	44.1 (4)
C27—C31—C30—Fe2	−59.16 (17)	C24—Fe1—C22—C23	−35.0 (2)
C32—Fe2—C30—C29	−171.3 (3)	C17—Fe1—C22—C23	123.9 (2)
C34—Fe2—C30—C29	107.1 (2)	C16—Fe1—C22—C23	165.4 (2)
C33—Fe2—C30—C29	151.32 (19)	C25—Fe1—C22—C21	39.7 (2)
C35—Fe2—C30—C29	66.9 (3)	C18—Fe1—C22—C21	−162.8 (2)
C31—Fe2—C30—C29	−119.1 (3)	C23—Fe1—C22—C21	116.9 (3)
C27—Fe2—C30—C29	−81.71 (18)	C20—Fe1—C22—C21	−41.3 (7)
C28—Fe2—C30—C29	−37.36 (16)	C19—Fe1—C22—C21	161.0 (3)
C32—Fe2—C30—C31	−52.2 (3)	C24—Fe1—C22—C21	81.9 (2)
C34—Fe2—C30—C31	−133.8 (2)	C17—Fe1—C22—C21	−119.1 (2)
C33—Fe2—C30—C31	−89.6 (2)	C16—Fe1—C22—C21	−77.7 (3)
C35—Fe2—C30—C31	−174.0 (2)		
